# The effect of arbuscular mycorrhizal fungi on biological activity and biochemical properties of soil under vetch growing conditions in calcareous soils

**DOI:** 10.1016/j.heliyon.2024.e24820

**Published:** 2024-02-01

**Authors:** Kader Burak, İbrahim Halil Yanardağ, María Dolores Gómez-López, Ángel Faz, Hamza Yalçin, Erdal Sakin, Emrah Ramazanoğlu, Aysel Bars Orak, Asuman Yanardağ

**Affiliations:** a Department of Soil Science and Plant Nutrition, Agriculture Faculty, Harran University, Sanliurfa, Turkey; b Soil Science and Plant Nutrition Department, Malatya Turgut Ozal University, Battalgazi, Malatya, Turkey; c Sustainable Use, Management and Reclamation of Soil and Water Research Group, ETSIA, Universidad Politécnica de Cartagena, Paseo Alfonso XIII, 48, 30203 Cartagena, Spain; d Republic of Türkiye Ministry of Agriculture and Forestry Directorate of Plant Protection Central Research Institute, Diyarbakır, Turkey

**Keywords:** Arbuscular mycorrhizal fungi (AMF), Vetch, Soil carbon–nitrogen–phosphorus cycle, Soil enzyme activity

## Abstract

Due to soils from arid regions with high lime and low organic matter content, farmers receive low yields along with high costs of agricultural inputs, which causes them to look for a solution. In this context, Arbuscular mycorrhizal fungi (AMF) have great potential to reduce fertilizer use by mediating soil nutrient cycles. However, little is known about studies of AMF inoculum on microbial biomass carbon (C), nitrogen (N), and phosphorus (P) cycling during vetch plant vegetation in calcareous areas. In this study, changes in soil biogeochemical properties related to soil C, N, and P cycling were investigated with five different AMF inoculations under vetch (common Vetch (CV; *Vicia sativa* L.) and Narbonne Vetch (NV; *Vicia narbonensis* L.) growing conditions. For the field study, a total of five different mycorrhizae were used in the experiment with the random plots design. AMF inoculation decreased the lime content of the soil, and the highest decrease was observed in NV with Glomus (G.) intraradices + G. constrictum + G. microcarpum inoculation (24.41 %). The highest MBC content was recorded in CV vetch G. intraradices (1176.3 mg C kg^−1^) and the highest MBN content in NV vetch G. intraradices + G. constrictum + G. microcarpum (1356.9 mg C kg^−1^). CAT activity of soils was highest in CV vetch G. intraradices (31.43 %) and lowest in NV vetch G. intraradices + G. constrictum + G. microcarpum (72.88 %), urease enzyme activity decreased in all treatments except G. constrictum + Gigaspora sp. and G. mosseae inoculations in CV. The highest DHG activity was detected in GF (15.72 %) AMFs in CV and GI (21.99 %) in NV. APA activity was highest in Glomus constrictum + Gigaspora sp. (23.33 %) in CV and Glomus fasciculatum (10.08 %) in NV. In CV plots, G. intraradices + G. constrictum + G. microcarpum (91.67 %) isolates had the highest and G. intraradices community had the lowest RC% (97.33 %) in mixed mycorrhiza species, while in NV plots G. fasciculatum inoculum had the highest and G. intraradices community had the lowest RC%. This study has important implications for the application of AMF for sustainable agriculture. When the results of the study were evaluated, the most effective AMF isolates in terms of C, N, and P cycles were G. constrictum + G. fasciculatum + Gigaspora sp. in Common vetch variety, and G. intraradices in Narbonne vetch variety.

## Graphical abstract

**Figure:**
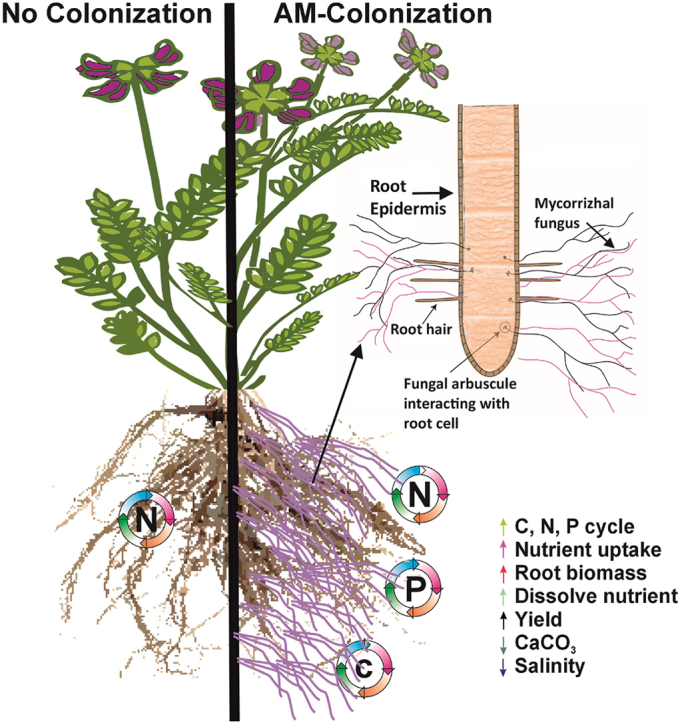

## 1 Introduction

Producing crops is a very laborious task for farmers, and they face the problems of being infertile poor soils, heat and drought stress, increasing production costs every year, and inadequate product prices. In this context, increasing fertilizer efficiency, reducing the amount of fertilizer, encouraging high rooting of the plant and easier and more efficient access to water are of vital importance. Mycorrhizae are the most widespread soil microorganisms forming a symbiotic relationship with more than 80 % of plants [1] and they can be found in diverse ecosystems worldwide [2]. Arbuscular Mycorrhizal Fungi (AMF) constitute the largest group among the different mycorrhizal groups, and they are AMFs that form mycorrhizal associations with plants colonized in the roots and establish a mutually beneficial relationship [3]. One of the benefits of symbiosis for plants is the resilience against stress factors such as metal toxicity, drought, and salinity, which enables some plant species to grow in harsh conditions. The enhanced water and nutrient availability provided by AMFs [4], even in nutrient-poor or dry soils, can positively affect other soil organisms, including bacteria and other fungi, by improving their nutrient supply and promoting their growth and activity, increasing the stress tolerance of plants, and extending root longevity [5]. Furthermore, AMF extraradical hyphae also play an important role in signaling and nutrient exchange by forming communal mycorrhizal networks between neighboring plants [6], and are therefore important functional groups for plant growth and soil quality maintenance [7]. AMFs such as G. intraradices increased the concentrations of organic acids such as proline and isocitrate [8]. G. constrictum had positive effects on photosynthetic pigments, gas exchange parameters, antioxidant enzymes, and nutrition of pepper plants grown under salt-stress conditions [9]. In banana plants inoculated with 2500 spores of G. microcarpum and F. mosseae, more than 80 % root colonization, increase in leaf chlorophyll content, leaf N, P, and K, significant decrease in soil pH, increase in soil available phosphorus and organic carbon were observed [10].

Organic matter positively affects the physical, chemical, and biological properties of soil [11], which is very important both because of this feature and because it is the largest terrestrial carbon reserve (about 0.68 Eg, i.e. 0.68 × 10^18^ g organic carbon) [12]. AMF hyphae are involved in soil C translocation and provide a key link in the terrestrial C cycle and thus play a crucial role in the global C cycle [13,14]. Indeed, AMF is an effective agent to improve carbon sequestration in the mechanism of translocation of C from high respiratory activity around the root to soil aggregates. They play a very important role in the hydrolysis of high-molecular-weight N-containing organic compounds of plant litter and soils to NH_4_^+^ in the regulation of N biogeochemical cycles in natural ecosystems [15]. They also exude large amounts of lytic enzymes and organic acids, which release recalcitrant organic and mineral nitrogen into the soil. These processes can bypass organic nitrogen mineralization by free-living decomposers, effectively short-circuiting soil–plant nitrogen cycling [15]. Also, AMFs are P activators that can accelerate the process of converting P into bioavailable forms through a series of chemical reactions and biological interactions [7].

In recent years, an increasing number of studies have recognized that the outcomes of plant-AMF interactions are on a continuum ranging from mutualism to parasitism, depending on the context in which interactions occur [16,17].

Vetches (Vicia sp.) is an important source of protein, minerals, vitamins, flavonoids, etc. in animal nutrition [18]. Narbonne vetch (V. narbonensis L.) ranks among the most important vetch species worldwide. Vetch crops affect the quantity and diversity of soil microorganisms due to their developed root system and low C:N ratio. Root secretions released by vetch plants, consisting of various organic compounds, can serve as a food source for soil microorganisms. These secretions promote the growth and activity of beneficial soil bacteria and fungi (such as nitrogen-fixing bacteria called rhizobia), increasing their populations in the rhizosphere [5,19]. Common vetch (Vicia sativa L.) is an annual legume grown as green manure and animal feed that provides rapid soil cover, and has the ability to increase soil moisture and organic matter content and reduce soil erosion [20]. These crops are particularly environmentally friendly, and their use is recognized as an important management practice with the potential to reduce dependence on mineral fertilizers and maintain soil organic matter content [21]. Since the availability of C substrates largely controls microbial growth in soil, green manure amendments promote microbial growth and activity in soil. Furthermore, legume-based green manures such as vetch and alfalfa are important sources of N in organic crop production [22].

Studying the response of soil microbial community and enzyme activities to warming provides a better understanding of soil biochemical processes under global warming [23]. The positive effects of AMF on improved soil fertility and plant community succession are well known [24]. However, the specific effects of AMFs on plant biomass, mycorrhizal colonization rate, soil microbial biomass, and soil enzyme activity may depend on factors such as soil conditions, environmental factors, and the cultivated varieties of plants.

There are many studies on the effects of different AMF isolate treatments on soil and plant properties. However, studies on the use of AMFs with different vetch varieties in semi-arid climatic zones are very limited. This study aimed to investigate changes in soil physicochemical properties and biochemical C, N, and P cycles in the soil (Calcareous)-plant (Common and Narbonne vetch)-root-microorganism (AMFs) ecosystem.

## 2 Material and method

The experiment was conducted under field conditions in Dipni (Döğer) Village of the Dicle district of Diyarbakır province in Southeastern Turkey (38°21′19″ N and 40°13′12’’E). The mean annual precipitation and mean annual temperature are 493.3 mm and 15.9 °C, respectively. The experiment was established in a 1.5 m × 6 m plot size under Narbonne vetch (*Vicia narbonensis* L.) and Common vetch (*Vicia sativa* L.) cultivation conditions. Vetch plant was sown (120 kg seed ha^−1^) with a 2 m margin between each plot. Both vetches were sown on October 16, 2020 according to the sprinkle sowing method. A total of five different mycorrhizae were used in the experiment and the experiment was carried out in 3 replications according to the random plots experimental design. The AMF isolates used in the experiment were added to the root zone on October 31, 2020 [25]. Mycorrhizal fungus inoculum was applied at a rate of 1000 spores/10 g soil. The experiment lasted 154 days. The mycorrhizal species used in the experiment and the experimental application plan are given in Table 1.

**Table 1:** Study area trial plan.

Narbonne Vetch	Common Vetch
1. Control, Co	1. Control, Co
2. GICM (G. Intraradices + G. Constrictum + G. Microcarpum)	2. GICM (G. Intraradices + G. Constrictum + G. Microcarpum)
3. GCF + GS (G. Constrictum + G. Fasciculatum + Gigaspora sp.)	3. GCF + GS (G. Constrictum + G. Fasciculatum + Gigaspora sp.)
4. GF (G. Fasciculatum)	4. GF (G. Fasciculatum)
5. GMS (G. Mosseae)	5. GMS (G. Mosseae)
6. GI (G. Intraradices)	6. GI (G. Intraradices)

The climate of the study area is continental, and agriculture is intensively practiced in the region. The soils of the study area belong to the sandy loam group according to the texture triangle. The soil is a Typic Xerorthents [26]. The average clay, silt, and sand contents of the soils were measured as 20.62, 12.37, and 67.01 %, respectively. The average EC of the soils is 388.33 μS cm^−1^ and there is no salinity problem. Soil reactions (pH) were slightly alkaline (7.57) and low in lime (8.05 %) (Table 2).

**Table 2:** Some properties of the soils of the study area.

Soil Properties	Mean	Min.	Max.	Std_Err.
Clay (%)	20.62	18.56	22.68	1.19
Silt (%)	12.37	10.31	14.43	1.19
Sand (%)	67.01	64.95	69.07	1.19
EC (μS cm^−1^)	388.33	380	396	4.63
Calcareous (%)	8.09	8.00	8.18	0.03
pH	7.75	7.70	7.80	0.03

The texture fractions of the soil samples were determined by the hydrometer method [27], the lime by Scheibler calcimeter [28], the soil reactions (pH) and electrical conductivity (EC) in saturation sludge (1:1 and 1:5 w/v, respectively) [29], and organic carbon according to the Walkley-Black wet burning method [29]. Determination of total nitrogen by Kjeldahl [30]. The amount of soluble C was detected by 0.5 M K_2_SO_4_ extraction and following 1 N K_2_Cr_2_O_7_ oxidation, absorbance was measured in a spectrophotometer at a 590 nm wavelength [31]. Microbial biomass carbon (MBC) content was determined according to the method of Vance et al. (1987). In the study, CO_2_–C emission measurements were made daily and weekly according to the amount of emission. CO_2_–C respiration was measured with the NaOH method [32].

The AMF isolates (G. intraradices, G. constrictum, G. microcarpum, and G. fasciculatum) used in the study were obtained from the Phytopathology laboratory of Diyarbakır Agricultural Control Research Institute, while G. mossea and G. intraradices were obtained from Adana Çukurova University Faculty of Agriculture Laboratory. Analyses related to mycorrhizal fungal isolates in the experiment (spore counting and root colonization) were carried out in the laboratory of GAP Diyarbakır International Research Institute Directorate and other parameters were carried out in the Soil Science and Plant Nutrition laboratory of Harran University Faculty of Agriculture.

### 2.1 Determination of enzyme activities in soil

For determinations of Dehydrogenase activity (DHG), 6 g of the soil was weighed out 0.06 g CaCO_3_, and 1 cm^3^ 3 % aqueous solution of 2,3,5-phenyl-tetrazolium chloride and 2.5 cm^3^ demineralized water were added to it successively. The samples were incubated for 24 h at 37 °C then measured by spectrophotometer at 485 nm wavelength [33].

Urease the intensity of the color formed after incubation of urease enzyme and soil samples with tris (hydroxymethyl) aminomethane (THAM) solution was determined in a spectrophotometer at 578 nm wavelength [34]. Five grams of moist soil was mixed with 20 ml acetate buffer (pH 5.2) and 100 mM р-NPP and incubated at 30 °C for 30 min. After incubation, 1 ml of CaCl_2_ and 4 ml of 0.2 M NaOH were added after incubation to terminate the reaction.

Catalase enzyme activity (mg KMnO_4_ kg^−1^) was determined by reduction in H_2_O_2_ by titration with 0.1 M KMnO_4_ after having shaken a 5 g soil sample in 100 ml distilled water for 30 min [35]. The alkaline phosphatase content of the soil was determined according to the method of Tabatabai and Bremner [36]. Soil was incubated with buffered (pH 6.5) sodium p-nitrophenyl phosphate solution and toluene at 37 °C for 60 min. Free p-nitro phenol content was measured using a spectrophotometer at 400 nm wavelength [36].

Soil microbial biomass C and N were estimated by extracting 10-g oven-dry equivalents of field-moist mineral soil samples in 0.5 M K_2_SO_4_ (1:4 w/v), by the chloroform fumigation-extraction method. Duplicate samples from each soil were placed inside 50-ml glass beakers. Samples were fumigated for 24 h at 25 °C in the dark [31].

### 2.2 Determination of AMF root colonization

The above-ground parts of the vetch plants were cut, and the root and root collar parts were slowly and carefully separated from the soil. The roots separated from the soil were washed thoroughly with water and the soil particles adhering to the roots were removed. Pieces of 0.5–1.0 g were taken from the root parts and placed in AFA (Ethyl Alcohol: Formaldehyde: Acetic Acid) fixation liquid for preservation and the roots were kept in this liquid until staining [37].

Roots preserved in AFA fluid were stained with lactophenol blue solution to determine the presence of mycorrhizal fungi and the percentage of colonization [37].

The Grid-Line Intersect Method was used to determine the percentage of AMF colonization in roots stained with lactophenol blue solution [38]. The stained capillary roots were cut into 1.0–1.5 cm lengths and approximately 0.5 g of these roots were sampled and distributed homogeneously in a plastic Petri dish divided into 1 cm^2^ areas. The Petri dish containing the root pieces was then examined under a stereomicroscope. In stereoscopic examinations, one button was pressed for each root segment perpendicular to the inter-sectional grids in the Petri dish, and two buttons were pressed together if AMF propagules (hyphae, vesicles, chlamydospores) were present in that vertical root segment. The percentage of AMF colonization was calculated with the following formula (Eq. 1);

Eq. 1 AMFColonization%=NumberofRootsColonizedwithAMFx100

TotalRootCount

AMF-free spores and sporocarps were separated from the soil by agitating 10 g < 2.00 mm aggregates of 100 g of mixed soil samples per experimental plot in water (no deflocculating agent was required), wet sieving, and filtration. Spores and sporocarps were counted under a stereoscopic microscope [39].

### 2.3 Statistical analysis

All statistical analyses were performed with the R environment “metabolomicsR” package [40]. Data were statistically analyzed using Levene's test for the assumption of equality of variance and the Shapiro-Wilk test for the assumption of normality (p < 0.05). The data were then analyzed using GLM (General Linear Model) and Tukey HSD multiple comparison test to determine whether there was a difference between the groups. Pearson correlation analysis was used to determine the relationships between variables. Clustering analysis is given with the heatmap for grouping the variables that are highly correlated with each other. In addition, network analysis was applied to better understand the pattern between variables. Data matrices normalized using log transformation are used for the debiased sparse partial correlation (DSPC). The algorithm is based on the decomposed graphical lasso modeling procedure and has been applied to explore the connections between a large number of variables using fewer samples [41]. Network degree and betweenness parameters were calculated for each node. Data are presented as mean and standard error. For all tests, significance was calculated at p < 0.05.

## 3 Results

### 3.1 Effect of AM fungal inoculum on some soil properties

According to the results of the analysis, no significant difference was observed between the interaction groups for clay, silt, sand, and pH (p > 0.05), while a significant difference was observed for lime and EC (p < 0.05) (Table 3). The data obtained in the post-trial analysis were compared with the Co group. GI, GCF + GS, GICM, GMS, and GF AMF isolates decreased the lime content of soils (23.35, 11.80, 11.43, 10.56, and 8.05 %, respectively). In terms of decreasing the lime content of soils, the highest decrease was realized by GI inoculum, and the lowest decrease was realized by GF. In NV-cultivated areas, the strongest AMF isolate was GICM, and the weakest was GI. Although there was no significant difference between the plants in terms of reducing the lime content of the soil in CV and NV cultivated soils, it was observed that the visible effect of the CV plant was higher.

**Table 3:** Some properties of soils after the experiment.

Vetch	Treatments	Lime	EC	pH	Clay	Silt	Sand
**Common Vetch**	**Control**	**8.05 ± 0.03 a**	**388.00 ± 5.00 a**	**7.60 ± 0.00 b**	**20.62 ± 1.19**	**12.37 ± 1.19**	**67.01 ± 1.19**
	**GICM**	7.13 ± 0.09 b	340.00 ± 10.00 c	7.60 ± 0.00 b	18.56 ± 1.19	12.37 ± 0.00	69.07 ± 1.19
	**GCF + GS**	7.10 ± 0.06 b	371.00 ± 5.00 abc	7.60 ± 0.00 b	20.62 ± 1.19	12.37 ± 0.00	67.01 ± 1.19
	**GF**	7.40 ± 0.06 b	356.00 ± 2.00 abc	7.60 ± 0.10 b	22.68 ± 1.19	8.25 ± 1.19	69.07 ± 1.19
	**GI**	6.17 ± 0.09 c	365.00 ± 5.00 abc	7.50 ± 0.00 b	18.56 ± 1.19	10.31 ± 0.00	71.13 ± 1.19
	**GMS**	7.20 ± 0.12 b	361.00 ± 13.00 abc	7.70 ± 0.00 b	19.24 ± 1.82	10.31 ± 1.19	70.44 ± 0.69
**Narbonne Vetch**	**Control**	**8.07 ± 0.03 a**	**378.00 ± 5.00 ab**	**7.70 ± 0.00 b**	**20.62 ± 2.38**	**10.31 ± 1.19**	**69.07 ± 1.19**
	**GICM**	6.10 ± 0.06 c	367.00 ± 11.00 abc	7.50 ± 0.00 b	21.31 ± 1.82	11.00 ± 0.69	67.70 ± 1.82
	**GCF + GS**	7.17 ± 0.09 b	371.00 ± 4.00 abc	7.70 ± 0.00 b	20.62 ± 1.19	10.31 ± 0.00	69.07 ± 1.19
	**GF**	6.20 ± 0.12 c	352.00 ± 4.00 BCE	7.60 ± 0.00 b	21.99 ± 1.82	8.94 ± 0.69	69.07 ± 1.19
	**GI**	7.30 ± 0.06 b	377.00 ± 3.00 a	7.60 ± 0.10 b	20.62 ± 2.06	8.25 ± 2.38	71.13 ± 1.19
	**GMS**	7.23 ± 0.03 b	364.00 ± 5.00 abc	7.60 ± 0.00 b	19.93 ± 0.69	10.31 ± 1.19	69.76 ± 1.82
	**p-value**	**<0.05**	**<0.05**	**>0.05**	**>0.05**	**>0.05**	**>0.05**

**GICM:** G. Intraradices + G. Constrictum + G. Microcarpum. **GCF + GS:** G. Constrictum (GC) + G. Fasciculatum (GF) + Gigaspora sp. (GS); **GF:** G. Fasciculatum; **GI:** G. Intraradices; **GMS:** G. Mosseae; *Different letters indicate significant differences (P* < *0.05) among means within each treatment.*

In CV-cultivated areas, GICM (17.32 %) and GCF + GS (4.38 %) AMF isolates decreased the soil EC value the most (p < 0.05). This decrease was 6.88, 3.75, 2.91, 1.85, 0.27 %, 3.75, 2.91 and 1.85 % for GF, GMS, GICM, GCF + GS, and GI AMF isolates inoculum to NV cultivated areas, respectively. The five AMF mycorrhiza isolates applied to the soil had no statistical effect on the pH and texture content of the soil (p > 0.05). Although the effect of AMF species on soil reaction in CV and NV cultivated areas was statistically insignificant (p > 0.05), a visible decrease was observed (Table 3).

### 3.2 Effect of AM fungal inoculum on soil C and N cycling

**Fig. 1:**
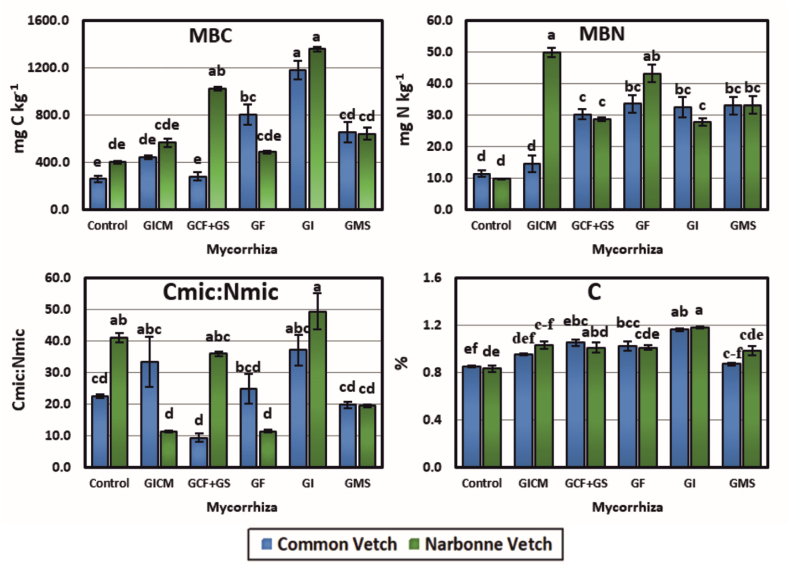
Effect of AM fungal inoculum on C and N cycling of soils. **MBC:** Microbial Biomass Carbon, **MBN:** Microbial Biomass Nitrogen, **Cmic:Nmic:** Microbial Biomass C/Microbial Biomass N, **C:** Organic C, **GICM:** G. Intraradices + G. Constrictum + G. Microcarpum. **GCF + GS:** G. Constrictum (GC) + G. Fasciculatum (GF) + Gigaspora sp. (GS); **GF:** G. Fasciculatum; **GI:** G. Intraradices; **GMS:** G. Mosseae. *Different letters indicate significant differences (P* < *0.05) among means within each treatment. Error bars denote standard error (n = 3).*

It was observed that AMF species affected both plant varieties and created a significant difference between soil MBC, MBN, C_MIC_: N_MIC,_ and OC% interaction groups (p < 0.05). All the AMF inoculum to CV significantly increased the amount of MBC compared to the Co group. This increase was highest in GI at 78.11 % and lowest in GCF + GS AMF(+) at 7.84 %. In NV plots, the highest increase was 70.56 % in GI and the lowest increase was 17.73 % in GF. In the case of CV and NV plant species without AMF, the most suitable species was NV, while in the case of AMF inoculants, the highest performance was obtained in CV_GI (Fig. 1).

GF, GMS, GI, GCF + GS, and GICM AMF isolates inoculated to CV and NV cultivated soils significantly increased the MBN contents of the soils compared to the Co group. The highest increase was found in GF (65.93 %) and the lowest increase was found in GICM (21.42 %) species in CV cultivated areas. In NV plots, the highest increase was recorded in GICM (80.44 %), and the lowest increase was recorded in GI (64.64 %). When the Co groups of both plant species were compared, CV gave the best response in terms of MBN. However, in the case of the AMF inoculum, the best result was realized in the NV vetch (Fig. 1).

C_MIC_: N_MIC_ ratios, which are significant indicators of decomposition and fragmentation, increased in GI, GICM, and GF inoculants in CV compared to the Co group, while decreasing in GMS and GCF + GS. In NV-cultivated plots, there was a decrease in GCF + GS, GMS, GF, and GICM species except for GI. When Co groups of CV and NV cultivated areas were compared, the best and fastest decomposition was found in the CV plant. According to AMF isolates, the fastest decomposition was observed in the GICM inoculant from NV (Fig. 1).

Organic C contents of CV and NV cultivated soils increased significantly compared to the Co group. GI inoculum to CV soils had the highest increase of 26.72 % and GMS had the lowest increase of 2.30 %. GI, GICM, GF, GCF + GS, and GMS inoculated to NV soils increased the OC content of soils by 29.66–15.31 %. When the Co group of CV and NV were compared, most OC was bound to the soil in CV-planted plots. In terms of increasing the OC content of soils, GI AMF inoculant was effective in both CV and NV cultivated areas (Fig. 1).

### 3.3 Effect of AM fungal inoculum on enzyme activity of soils

**Fig. 2:**
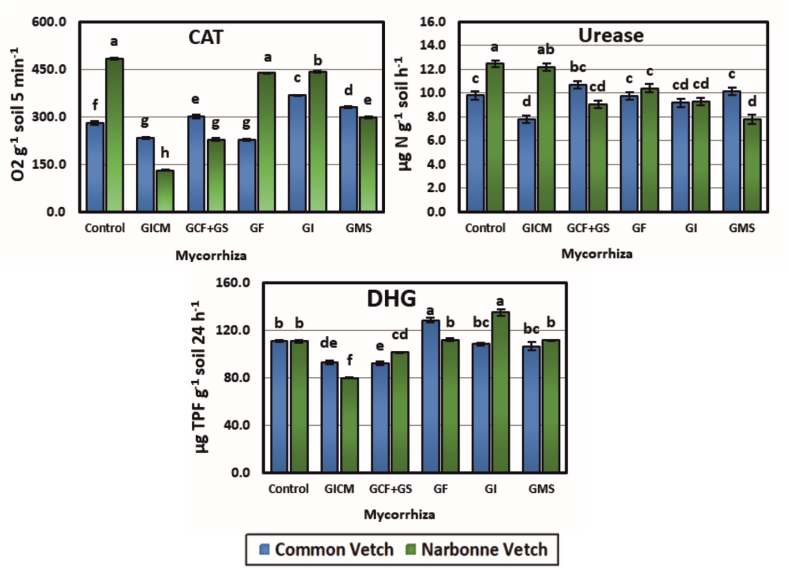
**Effects of AM fungal inoculum on** enzyme activities of soils **CAT:** Catalase enzyme activity, **DHG:** Dehydrogenase enzyme activity, **Urease:** Urease enzyme activity, **GICM:** G. Intraradices + G. Constrictum + G. Microcarpum. **GCF + GS:** G. Constrictum (GC) + G. Fasciculatum (GF) + Gigaspora sp. (GS); **GF:** G. Fasciculatum; **GI:** G. Intraradices; **GMS:** G. Mosseae. *Different letters indicate significant differences (P* < *0.05) among means within each treatment. Error bars denote standard error (n = 3).*

GI, GMS, and GCF + GS inoculations to CV cultivated areas increased the CAT enzyme activity of soils (23.91, 14.89, and 6.67 %) compared to the Co group, while GICM and GF inoculants decreased (16.79 and 18.93 %) (p < 0.05). All AMF isolates decreased the CAT enzyme activity of soils from NV-cultivated fields. The highest decrease was recorded in GICM (73.15 %) and the lowest in GI (8.69 %) inoculation. In CV-cultivated areas, the best performance was recorded in GI and the lowest in GF species. Although CAT activity decreased in NV-inoculated areas compared to the Co group, the best effect was observed in GICM, and the lowest effect was observed in GI. When the Co groups of CV and NV plant varieties were compared, the best performance was observed in the plots where the CV plant was planted (Fig. 2).

The urease enzyme activity of soils decreased in CV cultivated plots compared to the Co group except for GCF + GS and G_MS_ species. This decrease was lowest in GF (0.41 %) and highest in GICM (20.74 %). The increase in GCF + GS and GMS were 8.25 % and 3.74 %, respectively. In NV cultivated plots, urease decreased in all soil plots compared to the Co group (by 2.33–37.40 %). In CV plots, GCF + GS inoculations showed the best performance and GICM inoculations showed the weakest performance. In NV plots, the best activity was observed in G_ICM_ and the lowest in G_MS_ species. The urease enzyme activities and specific activity of various ponds planted with these plant species showed the following general trend: CV, GCF + GS > GMS > GF > GI > GICM, and NV, GICM > GF > GI > GCF + GS (Fig. 2).

Among the enzymes tested in CV cultivated plots, DHG was the most sensitive enzyme and increased with GF AMF inoculation (13.59 %), and decreased in all other treatments (2.43–16.85 %) compared to the Co group in the tested plots. In NV-cultivated fields, it decreased in GICM and GCF + GS inoculations (27.98 and 8.53 %) and increased in GI, GF, and GMS inoculations (18.03, 1.17, and 0.66 %, respectively). In terms of DHG enzyme activity, the best AMF performance was observed in the inoculations of GF in CV and GI in NV cultivated plots, while the worst performance was observed in GCF + GS in CV cultivated soil and GICM AMF strains in NV cultivated soil. When the Co groups of the two plants were compared, there was no superiority between them. However, in the study, the best performance was observed in NV plant + AMF isolates.

### 3.4 AM fungal inoculum effect on APA enzyme activity and P cycle

**Fig. 3:**
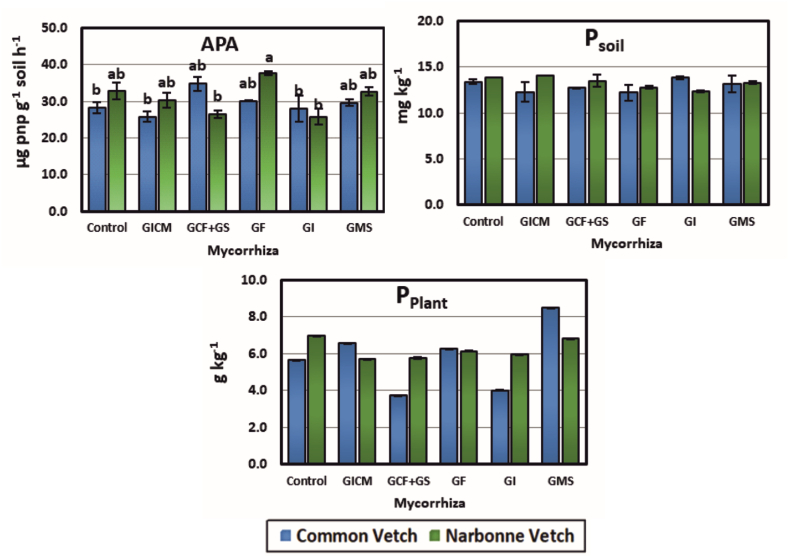
Effect of AM fungal inoculum on P cycle. **APA:** Alkaline phosphatase enzyme activity, **Psoil**: Plant Available Phosphorus in Soil, **Pplant**: Plant Available Phosphorus in Plant, **GICM:** G. Intraradices + G. Constrictum + G. Microcarpum. **GCF + GS:** G. Constrictum (GC) + G. Fasciculatum (GF) + Gigaspora sp. (GS); **GF:** G. Fasciculatum; **GI:** G. Intraradices; **GMS:** G. Mosseae. *Different letters indicate significant differences (P* < *0.05) among means within each treatment. Error bars denote standard error (n = 3).*

In CV plots, APA enzyme activity decreased in GICM and GI (8.64 and 0.57 %), while it increased in GCF + GS, GMS, and GF (18.92, 6.24, and 4.53 %, respectively). In NV, it increased with GF (13.11 %), while it decreased significantly with GMS, GICM, GCF + GS, and GI inoculations. When the Co groups of the plants were compared, the best result in terms of utilization was determined in the NV plant variety. The results of APA activity showed that the maximum level of hydrolytic processes was effective in both CV_GCF + GS and NV_GF mycorrhizal isolates (Fig. 3).

Soil available P content increased with G_I_ (by 2.97 %) and decreased with other AMFs (by 2.01–9.02 %) in CV fields compared to the Co group. In NV fields, it increased by 1.85 % with the G_ICM_ application and decreased by 2.46–10.49 % with other AMF applications. Although there was no significant difference between the Co groups of CV and NV plant species in terms of soil available P content (p > 0.05), the best result was observed in NV species. When AMF treatments were classified by plant, G_MS_ < G_CF_ + GS < G_ICM_ < G_F_ < G_I_ in CV plots in CV and G_I_ < G_F_ < G G_MS < CF_ + GS < G_ICM_ in NV plots were found to be the best working AMF inoculants in the soil (Fig. 3).

When the P content of plant tissues was compared with the Co group, the highest increase was observed in CV-cultivated plots with G_MS_ (33.53 %) treatment, and the highest decrease was observed with G_CF_ + GS (34.26 %) treatment. AMF inoculations applied to NV-cultivated plots decreased plant P content in all treatments. When the plants were compared in terms of plant P content, it was found that the NV plant was superior to the CV plant. Soil-applied AMF species showed that the CV plant was better than the NV plant in terms of P utilization (Fig. 3).

### 3.5 AM fungal inoculum effect on nutrients and root biomass

The ANOVA analysis results showed that a significant difference was observed between the interaction groups for AMF inoculums, RFW, plant N content (N_plant_), soil N content (N_soil_), soil nitrate (NO_3_^−^), and ammonium (NH_4_^+^) content (p < 0.05). In all plots planted with CV and NV, RFW showed a significant increase compared to the Co group. In CV plots, the highest increase was realized in GCF + GS (48.55 %) and the lowest increase was realized in the G_MS_ (3.85 %) treatment. In terms of root increase, AMF species GCF + GS > GI > GF > GICM > GMS provided more fresh residue. In NV plots, the highest increase was realized in G_I_ (43.60 %) and the lowest increase was realized in GMS (0.99 %). Among the AMF species applied to the soil in NV plots, GI > GICM > GF > GCF + GS > GMS provided more roots. In terms of fresh root weight, it was observed that the CV plant gave better results than NV. When plant species + AMF treatments were compared together, CV + AMF inoculations performed better in terms of RFW (Table 4).

**Table 4:** Effect of AM fungal inoculum on soil and plant N cycling and plant root biomass.

Alfalfa	Treatments	RFW (g)	N_plant_ (%)	N_soil_ (%)	NO_3_^−^ (%)	NH_4_^+^ (%)
**Common Vetch**	**Control**	121.50 ± 1.40 g	4.98 ± 0.03 abc	0.087 ± 0.003 de	0.92 ± 0.03 h	1.43 ± 0.03 d
	**GICM**	168.90 ± 3.32 de	3.34 ± 0.07 f	0.093 ± 0.003 bcde	3.55 ± 0.06 b	1.85 ± 0.03 c
	**GCF + GS**	236.15 ± 0.17 a	4.53 ± 0.03 d	0.087 ± 0.003 de	5.35 ± 0.03 a	2.22 ± 0.01 b
	**GF**	194.88 ± 2.25 c	4.17 ± 0.03 e	0.087 ± 0.003 de	1.94 ± 0.03 de	1.44 ± 0.03 de
	**GI**	210.03 ± 1.37 b	4.64 ± 0.18 cd	0.103 ± 0.003 BCE	1.21 ± 0.01 gh	1.46 ± 0.02 d
	**GMS**	126.36 ± 1.62 g	4.52 ± 0.02 de	0.057 ± 0.003 f	1.75 ± 0.29 ef	2.40 ± 0.03 b
**Narbonne Vetch**	**Control**	100.40 ± 2.90 h	5.30 ± 0.04 a	0.083 ± 0.003 e	1.41 ± 0.02 fg	2.36 ± 0.02 a
	**GICM**	156.50 ± 3.59 ef	4.62 ± 0.01 d	0.167 ± 0.003 a	1.51 ± 0.02 efg	1.38 ± 0.02 de
	**GCF + GS**	131.20 ± 1.31 g	5.11 ± 0.07 ab	0.107 ± 0.003 b	1.78 ± 0.01 def	1.89 ± 0.12 c
	**GF**	147.43 ± 1.49 f	4.83 ± 0.03 bcd	0.084 ± 0.003 e	2.86 ± 0.02 c	1.71 ± 0.01 c
	**GI**	178.01 ± 5.29 d	4.74 ± 0.08 cd	0.090 ± 0.000 cde	2.85 ± 0.03 c	1.19 ± 0.02 e
	**GMS**	101.40 ± 1.40 h	4.52 ± 0.03 d	0.100 ± 0.000 bcd	2.19 ± 0.01 d	2.39 ± 0.03 b
	**p-value**	**<0.05**	**<0.05**	**<0.05**	**<0.05**	**<0.05**

**RFW:** Root fresh weight, **N**_**soil**_**:** Soil total nitrogen, **N**_**plant**_**:** Plant total nitrogen, p < 0.05; at Significant, **GICM:** G. Intraradices + G. Constrictum + G. Microcarpum. **GCF + GS:** G. Constrictum (GC) + G. Fasciculatum (GF) + Gigaspora sp. (GS); **GF:** G. Fasciculatum; **GI:** G. Intraradices; **GMS:** G. Mosseae. *Different letters indicate significant differences (P* < *0.05) among means within each treatment.*

N_plant_ uptake of plants decreased in GI, GCF + GS, GMS, GF, and GICM species in CV cultivated plots. In NV-cultivated plots, it decreased with all treatments similar to CV. When two plants were compared in Co groups, the highest N_plant_ content was obtained in NV cultivated areas. In AMF treatments, the best result was obtained in CV vetch conditions (Table 4).

The N_soil_ contents of the soils increased in GI and GICM AMF treatments, decreased in GMS treatment, and did not change in GCF + GS and GF treatments in CV cultivated plots compared to the Co group. In terms of N_soil_ coverage of CV and NV cultivated soils, CV responded more favorably than NV. In the case of AMF species application, the NV plant showed the best response (Table 4).

NO_3_^−^ content of soils increased by 23.97–82.80 % with AMF applications in CV cultivated plots compared to the Co group. The highest increase was observed in GCF + GS and the lowest increase was observed in GI AMF treatments. In all plots planted with NV, NO_3_^−^ content increased according to the Co group, and the highest increase was observed in GF and the lowest increase was observed in GICM AMF types. When the Co groups were compared in terms of NO_3_^−^, the highest NO_3_^−^ content was measured in NV-cultivated soils. It was observed that the efficiency of NO_3_^−^ coverage with AMF applications to the soil was higher in CV plants (Table 4).

NH_4_^+^ contents of soils increased with all treatments in CV cultivated plots compared to the Co group, the highest increase was with G_MS_ (40.42 %) and the lowest increase was with G_F_ (0.69 %). NH_4_^+^ contents of NV cultivated soils decreased significantly in all treatments (19.92, 27.54, 41.53, and 49.5 % in GCF + GS, GF, GICM, and GI, respectively) except for G_MS_ (1.26 %). When the Co groups of both plants were compared, it was observed that NV had a higher NH_4_^+^ content. However, when AMF was inoculated, the CV plant was more effective in this direction (Table 4).

### 3.6 AM effect of fungi on % Root Colonization

Although there was a difference in all AMF inoculation treatments compared to Co treatments, this difference was found to have high colonization rates between +17 and 97 % (p < 0.05). In CV plots, the order of AMF communities in terms of root colonization rates was GICM > GCF + GS > GI > GF > GMS. In NV plots, the order of AMF communities in terms of root colonization rates is GF > GICM > GCF + GS > GMS > GI. The lowest root colonization performance rates among the AMF communities applied to both cultivated plots were observed in the GI community. It was revealed that the CV plant was superior in terms of root colonization performance rates with AMF inoculum to the soil (Table 5).

**Table 5:** Changes of root colonization with different AM fungal inoculum.

Plant	Treatments	Root colonization %
**Common Vetch**	**GICM**	91.67 ± 0.69 ab
	**GCF + GS**	71.67 ± 1.22 BCE
	**GF**	64.33 ± 0.66 cd
	**GI**	35.33 ± 0.12 ef
	**GMS**	46.00 ± 0.33 de
**Narbonne Vetch**	**GICM**	70.00 ± 0.00 bcd
	**GCF + GS**	45.33 ± 0.03 de
	**GF**	97.33 ± 0.49 a
	**GI**	10.00 ± 0.06 g
	**GMS**	17.33 ± 0.03 fg
	**p-value**	**<0.05**

p < 0.05; at Significant, **GICM:** G. Intraradices + G. Constrictum + G. Microcarpum. **GCF + GS:** G. Constrictum (GC) + G. Fasciculatum (GF) + Gigaspora sp. (GS); **GF:** G. Fasciculatum; **GI:** G. Intraradices; **GMS:** G. Mosseae. *Different letters indicate significant differences (P* < *0.05) among means within each treatment.*

### 3.7 Data grouping technique (heatmap)

**Fig. 4:**
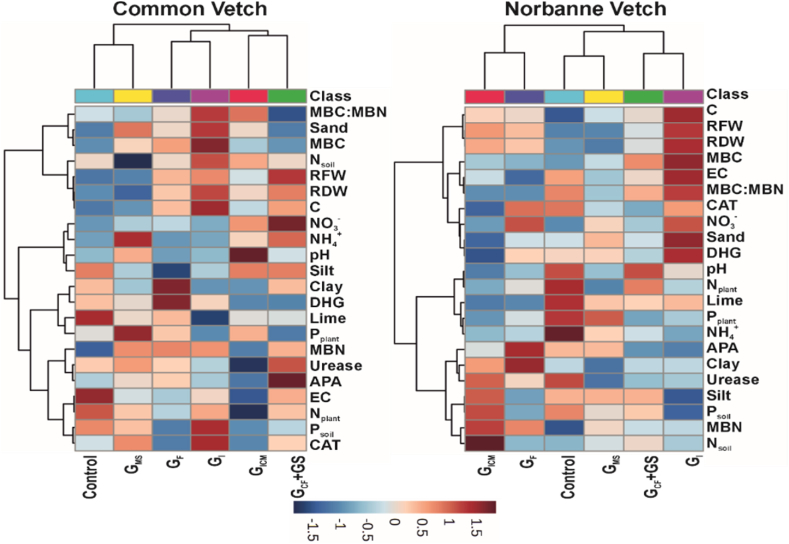
Correlation coefficients of some analyzed parameters (heatmap). **MBC**: Microbial Biomass Carbon; **MBN:** Microbial Biomass Nitrogen; **CAT:** Catalase enzyme activity, **DHG:** Dehydrogenase enzyme activity **APA:** Alkaline phosphatase enzyme activity; **RFW:** Root fresh weight; **RDW:** Root dry weight; **N**_**soil**_**:** Soil total nitrogen, **N**_**plant**_**:** Plant total nitrogen, **Psoil**: Plant Available Phosphorus in Soil, **Pplant**: Plant Available Phosphorus in Plant, **GICM:** G. Intraradices + G. Constrictum + G. Microcarpum. **GCF + GS:** G. Constrictum (GC) + G. Fasciculatum (GF) + Gigaspora sp. (GS); **GF:** G. Fasciculatum; **GI:** G. Intraradices; **GMS:** G. Mosseae.

Heat maps are a widely used visualization technique that can help to identify data efficiently and intuitively. This technique contributes to the understanding of the relationships between applications and measurements. In this paper, heat maps are used to visualize complex measurement data and to detect application changes. In the CV and NV examples in Fig. 4, all variables were standardized by equalizing their units [between −2 and +2] before analysis.

According to the results of cluster analysis of the data examined in Fig. 4, it is seen that 3 clusters were formed in both plants based on similarities. In the CV group, GICM-GCF + GS is divided into the first cluster with similarities, GF-GI into the second cluster with similarities, and Co-GMS into the third cluster with similarities. From the NV group, three separate clusters were formed according to the similarities of GI-GCF + GS, GMS-Co, and GICM-GF treatments.

On the other hand, according to the results of the analysis based on the measurements for CV, it is observed that 4 different clusters are formed when soil measurements are taken into consideration. In the first cluster, MBC: MBN, Sand, MBC, N_soil_, RFW, RDW, and C values are together. In the second cluster, NO_3_^−^, NH_4_^+^, pH and silt values come together. The third cluster contains Clay, DHG, lime, and P_plant_ values. Finally, in the fourth cluster, MBN, urea, APA, EC, N_plant_, P_soil,_ and CAT values are together (Fig. 4).

Furthermore, taking a broader perspective based on the measurements, 3 main clusters become more evident. The first cluster includes C, RFW, RDW, MBC, EC, MBC: MBN, CAT, NO_3_^−^, sand, and DHG, while the second cluster includes pH, N_plant_, lime, P_plant,_ and NH_4_^+^. The third and last main cluster includes APA, clay, urease, silt, P_soil_, MBN, and N_soil_. The results of this analysis provide us with important information to understand the similarities and differences between the variables (Fig. 4).

### 3.8 Data grouping technique. Network analysis of data

**Fig. 5:**
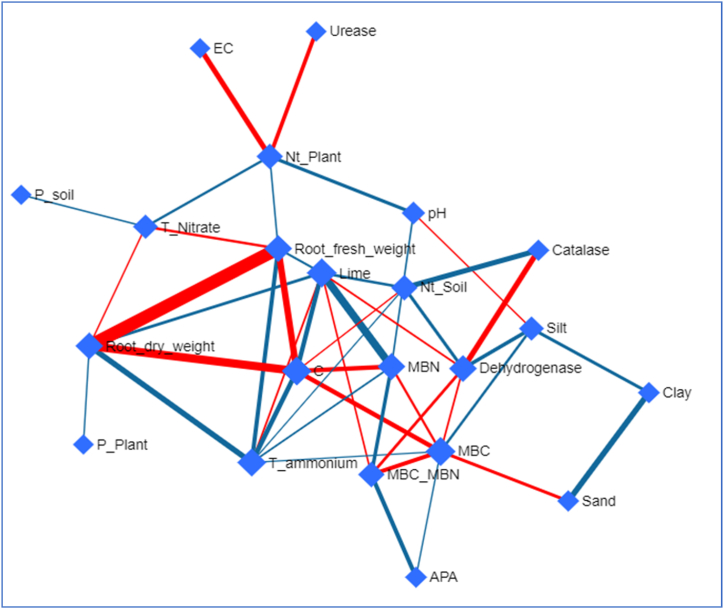
Network Analysis between the parameters analyzed in the study (red lines represent significant positive effect pathways; blue lines represent significant negative effect pathways (P < 0.05)). (For interpretation of the references to color in this figure legend, the reader is referred to the Web version of this article.)

According to the results of the Debiased Sparse Partial Correlation (DSPC) Algorithm, the MBN, and Lime tags have a high degree and betweenness centrality, indicating that they are well connected and influential within the network. NH_4_^+^ and C tags also have a relatively high degree, but their betweenness centrality is lower compared to other nodes. In the network pattern in Fig. 5, the small square boxes represent the nodes in the main network, while the positive and negative relationships between these nodes represent the edges. In this case, a blue link represents a negative correlation, a red link represents a positive correlation, the thinnest line represents the lowest correlation, and the thickest line represents the highest correlation.

Accordingly, when Fig. 5 is evaluated together with the correlation table (or Fig. 4), it can be seen that there is a strong positive correlation between RFW and RDW (r = 0.92), a strong positive correlation between RDW and C (r = 0.78), a moderate positive correlation between RDW and C (r = 0.64), strong negative between lime and MBN (r = −0.78), moderate negative between N_soil_ and CAT (r = −0.55), moderate positive between CAT and DHG (r = 0.53), moderate positive between C and MBN (r = 0.47), positive intermediate between EC and N_plant_ (r = 0.58), positive intermediate between urease and N_plant_ (r = 0.51), positive intermediate between RDW and C (r = −0.57), negative intermediate between C and NH_4_^+^ (r = −0.56), negative moderate correlation between RDW and NH_4_^+^ (r = −0.57), negative moderate correlation between RFW and NH_4_^+^ (r = −0.48), negative moderate correlation between MBC/MBN and APA (r = −0.53). In all correlation results, the interaction is at p > 0.01 level.

**Fig. 6:**
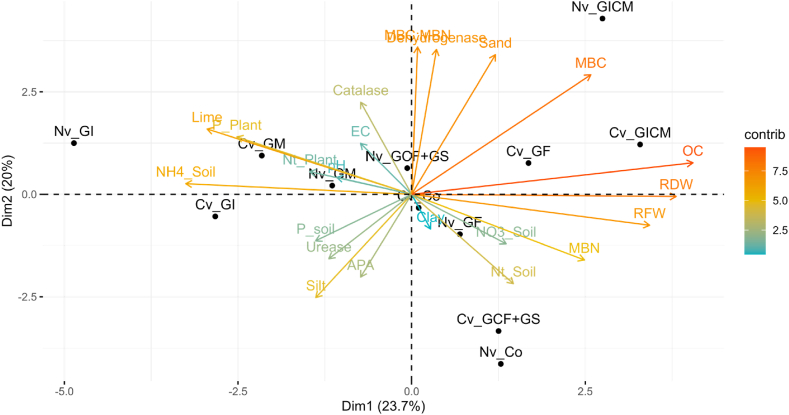
Biplot of principal component analysis (PCA) performed with AM fungal inoculum with the soil and plant parameters. **Cv:** Common vetch; **Nv:** Narbonne vetch; **RDW:** Root dry weight; **RFW:** Root fresh weight.

A dataset of 23 soil characteristics was analyzed using principal component analysis (PCA) to identify the main sources of variation in the data. The results of the PCA are presented in Fig. 6. The first principal component (PC1) accounts for 21.46 % of the variance in the data. The variables that have the highest loadings on PC1 are root dry weight, root fresh weight, t ammonium, time, dehydrogenase, MBC:MBN, MBC, and silica. These variables are considered to be the most important in explaining the overall variation in the soil characteristics.

The second principal component (PC2) explains 16.53 % of the variance in the data. The variables that have the highest loadings on PC2 are dehydrogenase, MBC:MBN, MBC, silt, sand, and pH. These variables are considered to be the most important in explaining the second most important source of variation in soil characteristics.

The third principal component (PC3) explains 13.96 % of the variance in the data. The variables that have the highest loadings on PC3 are Nt Plant, urease, P soil, APA, T Nitrate, catalase, Nt Soil, and clay. These variables are considered to be the most important in explaining the third most important source of variation in soil characteristics.

The fourth principal component (PC4) explains 9.99 % of the variance in the data. The variables that have the highest loadings on PC4 are APA, T Nitrate, catalase, Nt Soil, and EC. These variables are considered to be the most important in explaining the fourth most important source of variation in soil characteristics.

The fifth principal component (PC5) explains 8.87 % of the variance in the data. The variables that have the highest loadings on PC5 are P Plant, EC, and clay. These variables are considered to be the most important in explaining the fifth most important source of variation in soil characteristics.

Overall, the results of the PCA indicate that the soil characteristics can be summarized by a few main components. The first three components explain the majority of the variance in the data, and the variables that have the highest loadings on these components are likely to be the most important in understanding the variation in the soil characteristics.

## 4 Discussion

### 4.1 Effect of AMF on some soil properties

In this study, CV plant species were more effective than NV plant species in reducing the lime content of soils under both AMF and control conditions. These activities largely determine nutrient cycling and play an important role in the development of sustainable agriculture. However, related studies report largely different results for the effect of AMF on soil properties [42,43]. A decrease in the lime content of soils means a decrease in the pH value of the soil. Investigating how AMF stabilizes soil structure, Leifheit et al. [44] reported that AMF prefers soil pH close to neutral. Although the result of this study does not fully support the result of the present study due to the near-neutral soil pH, it shows that slightly alkaline soil pH can further enhance the functional performance of AMF in soil enzyme activity, and chemical and biological properties of soils. This means that there may be significant decreases in soil pH as a result of these applications for many years. This is because the decrease in the lime content of soils supports this, and the release of Ca^+2^ ions may also cause an increase in pH [43]. Therefore, the results of the present study are similar to those of Alguacil et al. [45]. Because they reported that soil pH may not be affected by AMFs.

Akhzari et al. [46] provided scientific evidence supporting the correlation between soil pH, 10.13039/501100000780EC, potassium, and AMF spore number, our results showed no significant positive correlation between soil pH and GICM in CV vetch, GCF + GS in NV vetch, 10.13039/501100000780EC, and GI AMF in NV vetch. These differences may be related to AMF species and behavior since some AM fungi prefer acidic soils while others do not [47]. In calcareous soils, AMFs have proved to be an effective method that can be used to increase plant growth and yield. The reduction of soil lime and EC by AMF applications is important for arid and semi-arid calcareous zone soils.

### 4.2 Effect of AMF on bio-chemical C and N cycles of soils

Fig. 1 shows that mycorrhizal roots create a sink demand for C and N. It is well known that increased populations of microorganisms in the soil increase the MBC and MBN content in the plant root zone, both due to the mineralization of organic wastes and because they are themselves a source of high-quality C and N. This increase in MBC and MBN increases plant nutrient availability by enhancing enzyme activity [48]. As increased plant biomass in the soil increases C availability, C allocation to AMF also increases and promotes AMF growth [49]. This C demand is supplied by C and transferred by the host plant through photosynthesis. Furthermore, AMF extramatric hyphae represent 10–80 % of soil microbial biomass, accounting for 15 % of soil organic C [50].

For efficient decomposition of organic matter by soil microorganisms, a soil C:N ratio of 10:1 is considered ideal. A balanced C:N ratio ensures that sufficient nitrogen is available to meet microbial demand during decomposition [51]. Generally, CV plots had lower C:N ratios and therefore higher mineralization (data not shown). However, the C:N ratio values of soils planted with NV cultivar vary in a significantly wider range (11–49), and thus the mineralization of the soil slows down the decomposition of organic matter [23,52]. In this study, this means that the NV plant is texturally more rigid and more resistant to decomposition than the CV plant. In addition, soils with a high C:N ratio have poor mineralization and nitrification, which is against nitrate formation and accumulation [53] because N mineralization and nitrification are positively correlated with soil NO_3_^−^-N content [54], which was supported by our results (Table 4).

### 4.3 Effect of AMFs on enzyme activity

Enzymes participate in many vital soil biochemical reactions and can have significant effects on soil fertility, strongly linked to AMF [55]. In our study, the effects of different AMFs on CAT, DGH, and urease enzymes were generally different from the control and these changes were statistically significant (p < 0.05). Urease is an agent that catalyzes the hydrolysis of soil-applied urea or existing urea to ammonia, causing the release of NH_3_ and pH increase, and is also the first step of the nitrification process [56]. In our study, there was a statistically significant correlation between urease and NH_4_^+^ (r = 0.38, weak positive), APA enzyme (r = 0.36, weak positive), and N_plant_ (r = 0.51, moderate positive) (Fig. 4). Based on this, it is thought that our study is consistent with the study conducted by Xiao et al. [57]. N_soil_ significantly affected soil urease, indicating that AMFs can affect soil nitrogen and have a significant relationship with soil nitrogen supply capacity. The analysis (DSPC) showed that soil microbial activity was affected by soil characteristics and AMF inoculation (Fig. 5).

In our study, AMF(+) inoculation significantly improved urease activities under the conditions under which both vetch cultivars were grown, indicating that AMF(+) inoculation contributed to the improvement of soil enzyme activity under vetch growing conditions (Fig. 2). It is consistent with AMF inoculation improving urease activities in the same soil and different plant species [58]. In particular, AMF enhanced urease activity by regulating extracellular enzymes [55] and facilitating the growth and development of microorganisms involved in soil N metabolism. The variation of urease activity by plant and AMF species has been demonstrated in many studies that vegetation types can modify the characteristics of soil microbial community structure and diversity [59,60].

When Co groups of CV and NV plants were compared in terms of CAT enzyme activity, NV had a higher activity. High CAT content in both plants was found in vetch plots where G_I_ inoculants were applied. The fact that CAT activity was lower in CV plant variety plots compared to NV suggests that this vetch species may be more tolerant to stress, and under extreme stress conditions, it is thought that plants increase microorganism activities in the root region by secreting sugars and other compounds from the phloem and that these microorganisms, whose activities increase, secrete CAT enzyme substrate to the root rhizosphere region. This is because the high activity of enzymes catalyzing the breakdown of H_2_O_2_ indicates that soil conditions are favorable for aerobic microorganism microflora [52]. CAT was significantly correlated with NH_4_ (r = 0.40, moderate positive), plant Nt (r = 0.42, moderate positive), N_soil_ (r = −0.55, moderate negative), DHG enzyme (r = 0.53 moderate positive) (Fig. 4). Environments such as climatic factors, soil, and spatial patterns influence edaphic microbial richness and community structure [61,62]. Plant species are the major factor in determining microbial diversity and community in the rhizosphere, resulting in different microorganism compositions for various species growing in the same soil [63]. In some cases, however, host variety may have more influence on microbial composition than soil and plant species [64].

Among the enzymes tested, DHG is the most sensitive and this enzyme decreased in all tested areas except for partial increases. The highest DHG activity was detected in AMF inoculations of G_F_ (13.59 %) in CV and G_I_ (18.03 %) in NV plots (Fig. 2). DHG activity was high in plots with high total C and total N utilization efficiency. The variability of DHG activity may be affected by soil C, N, lime, MBC, MBN, other enzyme activities, cultivation types, plant species, and genus. According to Burns [65], the effects of higher plants on soil enzymes depend on plant chemical composition, which can vary considerably between genera, species, and also between cultivars, even in the case of root exudates. Low DHG activities were observed in agriculturally cultivated soils, while Ostrowska and Porębska [66] reported higher activities of these enzymes in pasture soils. DHG activity of soils varies depending on the C:N ratio of soils. The relationship between enzyme activities and the C:N ratio confirms, among others, the importance of the quality of organic matter supplied by plants [66].

Statistically significant relationships were found between DHG activity and silt (r = −0.53 moderate negative), lime (r = 0.38, weak positive), N_soil_ (r = −0.52, moderate negative), Mmic:Nmic (r = 0.45, moderate positive), and Cmic (r = 0.48, moderate positive) (Fig. 4). Niemeyer et al. [67] state that the main negative impact on microbial indicators, and among them soil enzymes, is due to the limitation of plant re-establishment resulting in a low input of organic matter into the soil. In support of this, Patel and Patra [68] argue that the increase or decrease in DHG and APA activities is probably due to organic matter. DHG was estimated to vary because it is not always obvious in complex systems such as soils where the microorganisms and processes involved in the degradation of organic compounds are highly complex. From the above results and explanations, it is very clear that AMF can increase soil enzyme activity, which in turn can improve nutrient cycling.

In the present study, AMF treatments positively affected APA enzyme, soil, and plant P content (Fig. 3). This can be explained by the ability of AMF to convert inorganic phosphate into soluble forms through acidification, chelation, exchange reactions, and organic acid, H^+,^ and metabolite production processes [52]. In our study, AMF applications significantly decreased the lime content of soils (p < 0.05, Table 3), and the organic acid produced by AMF converted insoluble mineral phosphate into a soluble form [69]. This event is believed to be due to AMF hydrolyzing organic P to inorganic P through a mechanism linked to the production of enzymes called phosphatases [70]. However, it is worth mentioning that bacteria known as phosphate-solubilizing bacteria, which mineralize organic P and produce phosphatase, also have very important potential [71,72].

The reason for the lower P content of the soils compared to the Co group can be attributed to physical fixation and uptake by the plant due to high activity. This is also explained by the fact that plant P content and plant biomass were high in most AMF treatments. The findings obtained are in agreement with the findings of Huo et al. [73].

It is thought that the high P content of the soil in the areas where Co groups and some AMF(+) inoculants were applied in the study may have inhibited APA activity [74], because the high available P content of the soil may slow down AMF activity. This was clearly seen in the Co groups, explaining the weak activity in the CV with G_ICM_ and NV with G_I_ species (Table 4). In contrast to these views, Wei et al. [75] stated that there may be differences due to the variation in the regulatory gene system in the genotype.

### 4.4 Effect of AMF on some nutrients and plant biomass

It was reported that AMF colonization improved plant nutrients and uptake as well as below- and above-ground biomass [76]. In terms of the development of plant roots, it was observed that the CV plant had better root development and better nutrition. As can be seen from this, plant roots have a profound effect on soil nutrient dynamics. Understanding these effects is important for sustainable agriculture and soil fertility management. The AMF symbiosis results showed strong colonization effects on nutrient uptake for most of the other nutrients we measured, especially N_plant_, N_soil_, NO_3_^−,^ and NH_4_^+^ (Fig. 3, Fig. 4). The study by Lehmann and Rillig [77] supports this. However, the fact that some of these improvements (P_soil_, N_soil_, etc.) were lower than the control does not mean that there was little or no synergistic effect, but rather that the nutrients were taken up by the plant and used in its metabolic functions. This could also mean that the CV plant takes more nutrients from the soil and uses them in metabolic activities than the NV plant.

When the Co groups of the plants are compared, it is seen that the NV plant is more passive in the uptake of NO_3_^−^ and NH_4_^+^ from the soil. The high C content of the soil in which the CV plant was planted was found to improve soil N content (Fig. 1) due to its ability to retain N and reduce N losses through leaching [78]. With this improvement, the available N increased compared to the Co group and this increase could be attributed to the increased amount of subsoil biomass. Previous research has shown that plant-available N (NH_4_^+^ and NO_3_^−^) may decrease due to high C content, which stimulates microbial N immobilization [79]. In this study, similar to the study by Hu et al. [23], plant roots and AMF symbionts increased the ability of plants to obtain inorganic N from soil, increased the biomass of host plants, and reduced the NH_4_^+^ and NO_3_^−^ content of soil (Table 4).

Fall et al. [80] reported that the addition of AMF(+) inoculants resulted in higher crop yields. The best results in our study were observed in the increase in the amount of subsoil biomass of both legume crops with AMF inoculant (data not shown). This increase was 48.55 % in CV and 43.60 % in NV plants. This result is significant in all respects that the yield increase of subsoil biomass was realized without the use of fertilizers. The results presented in this study demonstrate the environmental benefits of using AMF to increase the productivity of legume crops. Similar results were reported for pineapple, where AMF inoculation and application of half the fertilizer dose promoted the highest levels of fruit mass and organoleptic variables [80]. An increase in sorghum yield was also reported by Ramadhani and Widawati [81], showing that a significant reduction of fertilizer in combination with AMF can reduce soil degradation and improve its quality. In this study, when the combination of GCF + GS inoculant was used for CV-cultivated areas and GI inoculant for NV-cultivated areas, the results showed that legume crops responded better to the inoculant (Table 4). AMF can therefore be seen as a good alternative to chemical fertilization, or at least reduce the need for large quantities of synthetic fertilizers (NPK) by half. Agricultural management practices based on AMF application can provide an economical, environmentally friendly, and sustainable way to improve soil fertility and yield.

### 4.5 AMF root colonization

The lowest root colonization rate was found in the GI community applied to CV and NV planted plots. It is thought that there may be some complementarity when the mixed community with the highest root colonization rates in CV plots is inoculated with GICM and GF with the highest root colonization rates in NV plots. However, it was observed that the mixed community and GF inoculations had a very similar potential synergistic effect on soil enzyme activities, plant nutrients, and some physical and chemical properties of soil, RFW, or nutrient uptake. These results showed that GICM and GF successfully colonized the rhizosphere of common and big vetch and moved effectively in the soil [82] Van der Heijden et al. [83,84] found that plants respond differently to certain AM fungal species. Therefore, a similar pattern of seasonal mycorrhizal colonization index should not be expected from experimental plants along the topographic gradient, especially when their root systems, growth periods and dependence on mycorrhizae to grow on nutrient-poor soils differ. Differences in colonized root length and plant biomass between CV and NV plants could be attributed to interactions between the growth rates of both fungi within roots and roots within the soil.

Although no fertilization including P was applied to the experimental plots, root colonization was high. Bars-Orak and Demir [85] investigated the effects of GI AMF species and different P doses in a similar region and climate. In their study, it was observed that AMF(+) plants were able to absorb more P than AMF(−) plants in foliar P analysis during the first flower formation period. Again, soil analysis showed that mycorrhizal plants were able to absorb sufficient P even when phosphorus was very low in the soil.

Some studies have reported that high phosphorus levels inhibit the colonization of AMFs [86]. Most soils used for agricultural purposes contain excess amounts of organic and inorganic P [87]. Much of this P can come from fertilizers applied to the soil for agricultural production [88]. When applying AMF to the soil, rather than applying P fertilizer, it is very important to make available the inorganic P present in the soil and fixed in any way. Thus, AMF can contribute to higher soil fertility and health in the root rhizosphere regions of the soil in arid and semi-arid regions, depending on root colonization and plant diversity.

In our study, the overall low root colonization of the NV legume plant was observed, which may be due to the low colonization rates of GI and GMS in the NV + AMF inoculant, which were similar in other plant varieties. For example, the average colonization percentages of a single G_MS_ strain ranged from 2.6 % to 27.0 % in a range of tomato cultivars [89]. Interestingly, the cultivar with the lowest colonization percentage still showed a significant increase in root dry weight of 43.60 % for Intradies (GI) and 0.99 % for Mosseae (GMS) in response to inoculation, while the root weight of the CV cultivar with the highest colonization percentage was significantly affected by G_I_ and G_MS_. This rather indicated that AMF community structure and mycorrhizal fungi were affected differently.

This study shows that regardless of the cause of low colonization of some AMF (+) inoculants, low colonization percentages can significantly affect plant performance (RFW weight) and that the magnitude of AMF effects is not necessarily related to colonization percentage. The results of the study are in agreement with the study conducted by Wang et al. [90].

### 4.6 Data grouping technique (network analysis-heatmap)

The functions and performance of roots in plants provide valuable information about the overall health of the plant and its response to environmental conditions [91]. Our study showed that the growth-promoting effects of different AMF inoculants applied to the soil where CV and NV plants were grown varied between the fresh weight and dry weight of the roots (p < 0.05). These results indicate that the plant growth-promoting effects of all the tested AMF inoculants were consistent between fresh and dry weights and that there was a very strong positive relationship between them (r = 0.9344, p = 0.0000; r = 0.987, p < 0.001) and studies support this relationship [91].

The moderate correlation between RDW and C (r = 0.57, p=<0.001) and RFW and C (r = 0.64, p=<0.001) is due to the fact that soil organic carbon is formed from the decomposition and breakdown of plant and animal tissue residues [56,[92], [93], [94]]. Of course, the amount of water contained in the tissue of the plant in RFW may be the reason why the C ratio differs slightly in dry weight. Increasing the amount of subsoil biomass (RDW) increases the C content. Many studies have shown that the positive relationship between RDW and C is strong [95]. A strong negative relationship (r = −0.78, p=<0.001) was found between MBC and MBN and lime; high lime content in soils may affect soil biological activities through its effects on the amount, structure, and distribution of soil organic matter. The lime content of soils can affect fungal and bacterial biomass differently [11,96]. Shah et al. [97] determined fungal and bacterial biomass separately and showed that lime content increased bacterial biomass only for a short time, while fungal biomass was not affected. Zelles et al. [98] suggested that increased lime slightly increased bacterial biomass, but significantly reduced fungal biomass.

There was a moderate negative correlation between soil N content and CAT enzyme activity (r = −0.55, p=<0.001). This suggests that the application of plant nutrients increases soil microbial biomass, but excessive fertilizer application reduces soil microbial biomass carbon [99], it has also been shown that fertilizer reduces microbial biomass by about −15 % [100]. Bargali et al. [101] showed that a moderate increase in nitrogen fertilizer application is beneficial to increase the carbon and nitrogen content of soil microbial biomass. There is a moderate positive correlation between CAT and urease enzymes (r = 0.53, p=<0.01). It is possible to see a similar moderate relationship between catalase and urease enzyme activities in other studies [102,103].

The presence of a moderate to positive correlation between C and MBN (r = 0.47, p=<0.01) is because microbial biomass acts not only as a C sink but also as an active driver of C and N transformation [104]. Although representing a small fraction of total soil C and N, microbial biomass plays a critical role in SOC mineralization [11,105]. There is a moderate positive correlation between urease and N_plant_ (r = 0.51, p=<0.01), indicating that plant N and urease activity have a significant positive correlation. It is one of the most important enzymes involved in the nitrogen cycle and has been positively correlated with many nutrients [106]. There was also a significant correlation between soil NH_4_–N content and urease activity [107].

The moderate negative correlation (r = −0.53, p=<0.01) between MBC:MBN and APA can be explained by the increased amount of organic C in the soil and thus lower microorganism activity. On the other hand, Liu et al. [108] reported that N application or the presence of sufficient nitrogen in the soil slowed down APA activity. Saha et al. [109], and Böhme and Böhme [110] reported that organic fertilization stimulated alkaline phosphatase activity, while P fertilizers had a negative effect. These contradictory results and interpretations found in the literature may be due to soil properties and/or different plant species studied.

## 5 Conclusion

AMF inoculum to the soil under Common Vetch and Narbonne Vetch cultivated areas decreased the Lime and EC content of the soil but had no significant effect on pH due to the short-term cycle. Agricultural management practices based on AMF application can provide an economical, environmentally friendly, and sustainable way to improve soil fertility and yield.

While positive effects on soil biochemical C, N, and P cycles were observed, it was observed that the rate of effect varied depending on the plant and AMF type. Especially G. constrictum + Gigaspora sp. in Common Vetch and GI AMFs in Narbonne Vetch were more effective than other treatments. The effects on soil enzyme activities (APA, CAT, DHG, Urease), MBC, and MBN were generally positive. The results of this study confirmed that AMF can release nutrients from complex materials by increasing soil enzyme activities. This study very clearly demonstrated that AMF could increase soil enzyme activity, which in turn can improve nutrient cycling.

In our study to make suggestions for future studies, it is seen that the highest root colonization rates can be achieved with the inoculum of Glomus intraradices + G. constrictum + G. microcarpum AMF isolates for Common Vetch and G. fasciculatum isolates for Narbonne Vetch.

## Data availability statement

The data that support the findings of this study are available on request from the corresponding author.

## Credit authorship contribution statement

**Kader Burak**: Methodology, Investigation, Formal analysis. **İbrahim Halil Yanardağ**: Writing - Review & Editing, Validation, Visualization. **María Dolores Gómez-López:** Writing - Review & Editing, Supervision. **Ángel Faz:** Writing - Review & Editing, Supervision. **Hamza Yalçin:** Validation, Software. **Erdal Sakin:** Writing - Review & Editing, Supervision, Project administration. **Emrah Ramazanoğlu:** Resources, Formal analysis, Investigation. **Aysel Bars Orak:** Resources, Formal analysis, Investigation. **Asuman Yanardağ:** Validation, Writing - Review & Editing.

## Additional information

No additional information is available for this paper.

## Declaration of competing interest

The authors declare that they have no known competing financial interests or personal relationships that could have appeared to influence the work reported in this paper.
